# Effects of gaps in priorities between ideal and real lives on psychological burnout among academic faculty members at a medical university in Japan: a cross-sectional study

**DOI:** 10.1186/s12199-017-0626-7

**Published:** 2017-04-04

**Authors:** Yuki Chatani, Kyoko Nomura, Saki Horie, Keisuke Takemoto, Masumi Takeuchi, Yukifumi Sasamori, Shinichi Takenoshita, Aya Murakami, Haruko Hiraike, Hiroko Okinaga, Derek Smith

**Affiliations:** 1 Department of Anesthesiology, Saitama National Hospital, National Hospital Organization, Suwa2-1, Wako-city, Saitama 351-0102 Japan; 2 Department of Hygiene and Public Health, Teikyo University School of Medicine, Kaga2-11-1, Itabashi-ku, Tokyo, 173-8605 Japan; 3 Support Center for Women Physicians and Researchers, Kaga2-11-1, Itabashi-ku, Tokyo, 173-8605 Japan; 4 Teikyo University Graduate School of Public Health, Tokyo, Japan; 5 Department of Value & Decision Science, Tokyo Institute of Technology, Tokyo, Japan; 6 The Impulsing Paradigm Change through Disruptive Technologies Program, (ImPACT), founded by Cabinet Office, Government of Japan, Tokyo, 102-0076 Japan; 7 Department of Obstetrics and Gynecology, Teikyo University School of Medicine, Kaga2-11-1, Itabashi-ku, Tokyo, 173-8605 Japan; 8 Department of Law, Faculty of Law, Teikyo University, Tokyo, Japan; 9 College of Public Health, Medical and Veterinary Sciences, James Cook University, Townsville, Australia

**Keywords:** Academic professions, Children Psychological burnout, Priority gap between ideal and real lives, Social support

## Abstract

**Background:**

Accumulating evidence from medical workforce research indicates that poor work/life balance and increased work/home conflict induce psychological distress. In this study we aim to examine the existence of a priority gap between ideal and real lives, and its association with psychological burnout among academic professionals.

**Methods:**

This cross-sectional survey, conducted in 2014, included faculty members (228 men, 102 women) at a single medical university in Tokyo, Japan. The outcome of interest was psychological burnout, measured with a validated inventory. Discordance between ideal- and real-life priorities, based on participants’ responses (work, family, individual life, combinations thereof), was defined as a priority gap.

**Results:**

The majority (64%) of participants chose “work” as the greatest priority in real life, but only 28% chose “work” as the greatest priority in their conception of an ideal life. Priority gaps were identified in 59.5% of respondents. A stepwise multivariable general linear model demonstrated that burnout scores were associated positively with respondents’ current position (*P* < 0.0018) and the presence of a priority gap (*P* < 0.0001), and negatively with the presence of social support (*P* < 0.0001). Among participants reporting priority gaps, burnout scores were significantly lower in those with children than in those with no children (*P*_interaction_ = 0.011); no such trend was observed in participants with no priority gap.

**Conclusions:**

A gap in priorities between an ideal and real life was associated with an increased risk of burnout, and the presence of children, which is a type of “family” social support, had a mitigating effect on burnout among those reporting priority gaps.

## Background

Burnout is a disastrous condition characterized by physical, emotional, and mental exhaustion that can be caused by emotional damage [1]. Accumulating evidence from medical workforce research indicates that poor work/life balance and increased work/home conflict induce psychological distress, characterized by anxiety, stress, broken relationships, depression, and/or burnout [2–4]. Burnout syndrome can affect individuals in a variety of professions that require intense interaction with other people, such as police officers, social workers, and nurses. Its consequences may be particularly important in the medical field; it has been associated with an increased risk of medical errors [5], suboptimal patient care [6], and reduced patient satisfaction. Burnout can also affect medical professionals’ personal lives, including relationships and activities outside the professional domain, often propelling them to consider early retirement [7]. Although associated factors and methods of resolving burnout have been discussed for a long time, no clear conclusion has been reached.

In this study, we focused on academic faculty members at a single medical university in Tokyo, Japan. As noted recently in Nature, young researchers are often excluded from Japanese universities and the number of young faculty members at these institutions is declining [8]. In addition, the number of publications originating from Japanese universities, companies, and organizations decreased by 4.3% between 2006 and 2010, whereas the numbers of such publications originating from the UK and Germany increased by 12.7 and 15%, respectively, during the same time period [9]. In Japan, obtaining academic employment related to medicine is known to be highly competitive, and the work required to retain such a position is known to be demanding. Thus, we hypothesized that the priority gap between conceptions of an ideal life and real life would create psychological distress in this population. Although few studies have examined this issue, one survey of psychiatrists working at medical schools throughout Japan demonstrated that difficulty with work/life balance and less work-environment satisfaction were associated significantly with greater emotional exhaustion [8]. Hence, the purpose of this study was to investigate the effect of an ideal-/real-life priority gap on burnout among academic faculty members at one medical university.

## Methods

### Participants

This cross-sectional survey was conducted at one university and one affiliated hospital from January to March 2014. We consecutively recruited 1189 faculty members, 1235 medical staff members, and 266 clerks. Of those who returned questionnaires, we excluded hospital workers (i.e., nurses, pharmacists, technicians, and clerks). Thus, the final sample consisted of 330 academic faculty members (228 men, 102 [31%] women), 54% of whom were aged 50–65 years (response rate, 27.8%). The participants worked in the following departments: Medicine (*n* = 94 [28.5%]), Medical Technology (*n* = 67 [20.3%]), Science and Engineering (*n* = 36 [10.9%]), Pharmacology (*n* = 28 [8.5%]), Economics (*n* = 26 [7.9%]), Literature (*n* = 22 [6.7%]), Education (*n* = 17 [5.2%]), Law (*n* = 9 [2.7%]), other (*n* = 17 [5.2%]), and not specified (*n* = 14 [4.1%]. The institutional review board of Teikyo University School of Medicine approved this study (no. 13–1310).

### Measures

#### Outcome

The outcome of interest was burnout, measured by a 17-item inventory, developed originally for the assessment of workplace burnout by Maslach and Jackson [10] and translated into Japanese by Tao and Kubo [11]. Item responses are structured by a five-point Likert scale. Total scores range from 17 to 85, with higher scores indicating a greater risk of burnout.

Burnout was defined as a syndrome characterized by lack of interest in/enthusiasm for work (emotional exhaustion), a tendency to treat people as if they were impersonal objects (depersonalization), and a sense that one’s work is not meaningful or important (low sense of personal accomplishment).

#### Exposure

Priorities in a conception of ideal life and in real life were measured by asking participants, “What do you want to prioritize in your life?” and, “What do you prioritize in real life?,” respectively. Response choices included work, family, individual life, and combinations thereof. We also asked participants, “What do you want your partner to prioritize in your ideal life?” and, “What does your partner prioritize in your real life?” Differences in responses about ideal and real lives were used to define priority gaps.

### Covariates

Covariates used in this study were gender, age, marital status, presence/absence of children, partner’s occupation (employed/homemaker or unemployed), current position (professor/associate professor/lecturer/assistant professor/instructor), and household income (upper/upper middle/middle/lower middle/lower). Household was measured by asking “Suppose if annual individual income is classified into five ranks, which categories do you think your income falls in?” We also asked about the average number of hours per day spent working at the workplace and in the household, and about social support. Social support scores were calculated using the 15-item Social Support Questionnaire originally developed by Sarason [12]. Questionnaire scores range from 15 to 75, with higher scores indicating more support.

### Statistical analyses

Baseline characteristics and working conditions of men and women were compared using the *t*-test for continuous variables and the chi-squared test for categorical variables. We estimated regression coefficients and standard errors of each variable associated with the total burnout score using univariate and multivariate general linear models. Stepwise model selection was performed using the SAS glmselect procedure, and the final model was determined using Akaike’s information criterion. Selection was performed in three steps: the initial model included all explanatory variables, the second model included selected variables from the first model and all potential interaction terms between these variables (forced inclusion), and the final model included selected variables and interaction terms without forced inclusion. All analyses were performed using SAS software (version 9.3; SAS Institute Inc., Cary, NC, USA), with a two-tailed significance level of *P* < 0.05.

## Results

Table 1 shows participants’ baseline characteristics and working conditions according to gender. Women were more likely than men to be single (*P* = 0.008), to have employed spouses (*P* < 0.001), to report personal and partner priority gaps (*P* = 0.002 and *P* < 0.001, respectively), and to work longer at the workplace and at home (*P* = 0.001). Women were also less likely to have children (*P* = 0.002) and to hold positions higher than lecturer (*P* < 0.001).

**Table 1:** Baseline characteristics and working conditions according to gender

	Total (%) *n* = 328	Women (%) *n* = 102	Men (%) *n* = 228	*P*-value
Marital status				0.008
Married	262 (79)	72 (71)	190 (83)	
Single (Including Divoced or Widowed)	68 (21)	30 (29)	38 (17)	
Presence of children				0.002
No	118 (36)	49 (48)	69 (30)	
Yes	211 (64)	53 (52)	158 (70)	
Age group				0.077
20s or 30s	66 (20)	27 (27)	39 (17)	
40s	84 (26)	28 (27)	56 (25)	
50s or more	179 (54)	47 (46)	132 (58)	
Spouse’s employment				<0.001
Employed	164 (63)	69 (96)	95 (50)	
Unemployed	98 (37)	3 (4.2)	95 (50)	
Current position				<0.001
Lecturer or upper	252 (76)	65(64)	187 (82)	
Assistant or lower	78 (24)	37 (36)	41 (18)	
Household income				0.350
Upper	168 (51)	46 (46)	122 (53)	
Middle	119 (36)	42 (42)	77 (34)	
Lower	41 (13)	12 (12)	29 (13)	
Gap of priority between ideal and real lives				0.007
No	133 (41)	29 (28)	104 (46)	
Yes	195 (59)	73 (72)	122 (54)	
Gap of priority in a partner between ideal and real lives				<0.001
No	125 (47)	21 (29)	104 (54)	
Yes	140 (53)	52 (71)	88 (46)	
Work hours (means ± SD)				0.021
	9.7 ± 2.1	9.3 ± 1.8	9.8 ± 2.2	
Hours of house chores (means ± SD)				0.001
	11.3 ± 2.3	11.9 ± 2.0	11.1 ± 2.3	
Social support (means ± SD)				0.180
	54.2 ± 12.8	55.6 ± 12.9	53.5 ± 12.8	

Any categories which does not become 100% has missing values

Household income was grouped into three categories (i.e., “upper/upper middle”, “middle”, “lower middle/lower”

Table 2 shows data on discordance between ideal- and real-life priorities. Responses from 195/328 (59.5%) participants revealed priority gaps. Significantly more participants (*n* = 209 [64.0%]) chose “work” as the greatest priority in real life compared to the situation in an ideal life (28.0%). This result was not changed by the exclusion of “do not know” responses from the analyses.

**Table 2:** Contingency table of priority in between real and ideal life

		Priority in real life								
		work	family	individual	work & family	work & individual	family & individual	three of work, family, and individual	do not know	total
Priority in ideal life	work	58 (27.8)	0	0	0	1 (7.7)	0	0	0	59
	family	9 (4.3)	1 (12.5)	0	2 (2.6)	0	0	0	0	12
	individual	1 (0.5)	0	0	0	2 (15.4)	0	0	0	3
	work & family	77 (36.8)	7 (87.5)	0	55 (70.5)	0	0	0	2 (33.3)	141
	work & individual	11 (5.3)	0	1 (100)	2 (2.6)	5 (38.5)	0	0	0	19
	family & individual	5 (2.4)	0	0	0	1 (7.7)	0	0	1 (16.7)	7
	three of work, family, and individual	44 (21.1)	0	0	19 (24.4)	4 (30.8)	1 (100)	12 (100)	1 (16.7)	81
	do not know	4 (1.9)	0	0	0	0	0	0	2 (33.3)	6
	total	209	8	1	78	13	1	12	6	328

Table 3 shows the results of the univariate general linear model for burnout. Significant variables in this model were gender (*P* = 0.036), marital status (*P* = 0.001), presence of children (*P* < 0.001), age group (*P* = 0.003), current position (*P* < 0.001), household income (*P* < 0.001), personal and partner priority gaps (*P* < 0.001 and *P* = 0.022, respectively), and workplace/household working hours (*P* = 0.004).

**Table 3:** General linear models of an effect of covariates on burnout, both univariate and multivariate models

	Univariate			Multivariate (Stepwise model)					
				Model 1 *n* = 302、R2 = 0.23			Model 2 *n* = 301、R2 = 0.25		
	Point estimate	SE	*P*-value	Point estimate	SE	*P*-value	Point estimate	SE	*P*-value
Sex			0.033						
Women	2.8	1.3							
Men	-	-							
Marital status			0.002						
Married	4.5	1.5							
Single (including Divoced or Widowed)	-	-							
Presence of children			<0.001			0.005			0.960
No	4.9	1.2		3.4	1.2		0.1	1.8	
Yes	-	-		-	-				
Age group			0.005						
20s or 30s	4.1	1.6							
40s	3.9	1.4							
50s or more	-	-							
Spouse’s employment			0.380						
Unemployed	1.1	1.3							
Employed	-	-							
Current position			<0.001			0.014			0.002
Assistant or lower	5.9	1.4		4.5	1.4		4.3	1.4	
Lecturer	-	-		-	-				
Household income									
Lower	8.7	1.9	<0.001						
Middle	2.2	1.3							
Upper	-	-							
Gap of priority between ideal and real lives			<0.001			<0.001			<0.001
Yes	7.0	1.2		6.2	1.1		10	1.9	
No	-	-		-	-		-	-	
Gap of priority in a partner between ideal and real lives			0.022						
Yes	2.8	1.2							
No	-	-							
Work hours			0.300						
More than 8 h	2.0	1.9							
Less than 8 h									
Hours of house chores (means ± SD)	-	-	0.003						
More than 8 h	4.2	1.4							
Less than 8 h	-	-							
Social Support	−0.28	0.045	<0.001	−0.22	0.043	<0.001	−0.21	0.043	<0.001
Statistical interaction									
Presence of children × Presence of WLB gap							−5.9	2.3	0.011

In the multivariate analysis, including all explanatory variables (model 1), the presence of children, current position, and priority gap were significant. In model 2, constructed by forced inclusion of these variables and all potential interactions between them, the priority gap, presence of children, current position, and interaction between the priority gap and presence of children were significant. In the final model, which included these variables and interaction terms and was analyzed by stepwise selection without forced inclusion, the same variables were significant in both model 1 and model 2. Burnout scores were ten times higher among respondents reporting priority gaps than among those reporting no gap (*P* < 0.001; Table 3).

**Fig. 1:**
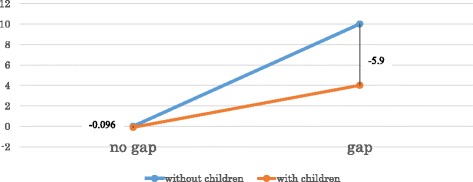
An effect of WLB gap on burnout (with or without children)

Among participants reporting priority gaps, burnout scores were significantly lower in those with children than in those with no children (*β* = −5.9, *P*_interaction_ = 0.011). Burnout scores were similar among those reporting no priority gap, with and without children (*β* = −0.096, *P* = 0.96). (Fig. 1).

## Discussion

Approximately 60% of participants in this study reported discordance between ideal- and real-life priorities. Burnout scores were associated positively with current position and this priority gap, and negatively with the presence of social support. Among participants of both genders reporting priority gaps, the presence of children had a mitigating effect on the burnout score.

In general, the presence of children is thought to be a cause of work/home conflict, which can provoke burnout [13]. Previous researchers [14] have found that perceived social support can affect individuals’ emotional well-being, in agreement with our results. The perceived social support factors of “family” and “friends” have consistently shown the strongest associations with symptomatology, such as depressed psychological state, in college students (*n* = 549) and psychiatric outpatients (*n* = 156) [15]. Umene et al. [8] demonstrated that social support had a mitigating effect on burnout, which was associated significantly with poor work/life balance and the number of nights worked per month. Our finding that the presence of children mitigated psychological burnout suggests that emotional support from children is a type of “family” social support, which appears to have a good influence on the mental health of faculty researchers, helping to alleviate work-related psychological stress.

Previously, we demonstrated that early pregnancy age among female doctors was associated with less likelihood of attaining a board specialty or DMSc [16], suggesting that those whose careers are not yet established should use caution when deciding whether to have a child at an earlier age. The gender division of labor is embedded strongly in the mindset of Japanese culture, and finding a balance between career development and child rearing is very difficult, especially for women [17]. In addition, this study found that higher position like “professor” is negatively associated with burnout. Because we confirmed that women were less likely to be in higher position, female faculties and especially those who had children are thought to be vulnerable to burnout. In this regard, having a child can be a double-edged sword; it negatively affects young female health professionals, who are required to study and rear children at the same time [16], but it may positively affect workers with perceived priority gaps.

Psychological burnout is a known risk factor for quitting one’s job or choosing early retirement, especially among women. Solutions for psychologically vulnerable workers that have been presented in the literature include mentorship and coaching, recently considered to be among the most powerful tools to help keep female health professionals working [13]. In addition, although very few studies have investigated the usefulness of “work/life balance interventions”, Fortney et al. [18] reported that a self-efficacy intervention involving training in mindfulness practice positively affected job satisfaction, quality of life, and compassion in the context of work; alleviated burnout and stress; and amplified resilience and empathy.

Due to the gender division of labor, the acquisition rate of child-care leave differs dramatically between men and women (1.7 and 86%, respectively) [19]. Johhanson et al. [20] pointed out the importance of preparation classes for reassurance and the acquisition of child-raising skills for fathers, and argued that policy makers and health-care providers should offer this kind of support, as it benefits not only men but also their children and partners, and ultimately helps to encourage egalitarian practices at home and work. Even in Sweden, one of the most advanced countries in the world in terms of welfare, fathers’ participation in child rearing remains a concern. Despite the inherent difficulties, policy makers in Japan should make further efforts, using multidirectional approaches to support female and male workers’ ability to continue work and develop careers.

This study has several limitations. First, it involved a small sample of participants from a single private medical university. Our participants were likely to have heavy workloads, which is often the case in the highly competitive work environment of academic medicine in Japan. Compared with respondents in a survey conducted by the Japanese Cabinet Office using the same items as in this study [21], more of our participants chose “work” (49% vs. 64%) and fewer chose “family” (19% vs. 2.5%) as the greatest real-life priority. Hence, our results require careful interpretation.

Second, our analyses examined only overall burnout scores. Many studies of burnout have involved the analysis of associations of individual burnout factors (emotional exhaustion, depersonalization, and low personal accomplishment) with covariates. We conducted such analyses, but they yielded no remarkable finding beyond the results of overall analyses. In addition, because such detailed analysis makes interpretation of the results complicated and difficult, we presented data only on overall burnout scores. Third, our result might have been different according to types of medical profession like medical doctors, nurses, or other health professions. This speculation however, could not be verified due to insufficient numbers of the responses.

## Conclusion

We found that burnout scores were higher among participants reporting ideal-/real-life priority gaps than among those with no gap, in analyses adjusted for covariates. In addition, the presence of children had a mitigating effect on burnout. Child rearing is time consuming, especially when the child is small, but the presence of children later in life may mitigate psychological stress among parents in the workforce. The results of this study imply that the presence of children plays an important role in alleviating psychological stress not only among female but also among male faculty members.
